# *SERPINA1* gene identified in RNA-Seq showed strong association with milk protein concentration in Chinese Holstein cows

**DOI:** 10.7717/peerj.8460

**Published:** 2020-02-24

**Authors:** Cong Li, Wentao Cai, Shuli Liu, Chenghao Zhou, Hongwei Yin, Dongxiao Sun, Shengli Zhang

**Affiliations:** 1 College of Animal Science and Technology, Northwest A&F University, Yangling, Shaanxi, China; 2 Department of Animal Genetics and Breeding, College of Animal Science and Technology, China Agricultural University, Beijing, China

**Keywords:** Candidate gene, *SERPINA1*, Milk protein trait, Association analysis, Linkage disequilibrium, Haplotype, Phenotypic variance, Molecular marker, Selective breeding, Dairy cows

## Abstract

The detection of candidate genes and mutations associated with phenotypic traits is important for livestock animals. A previous RNA-Seq study revealed that *SERPINA1* gene was a functional candidate that may affect milk protein concentration in dairy cows. To further confirm the genetic effect of *SERPINA1* on milk protein traits, genetic polymorphisms were identified and genotype-phenotype associations were performed in a large Chinese Holstein cattle population. The entire coding region and the 5′-regulatory region (5′-UTR) of *SERPINA1* was sequenced using pooled DNA of 17 unrelated sires. Association studies for five milk production traits were performed using a mixed model with a population encompassing 1,027 Chinese Holstein cows. A total of four SNPs were identified in *SERPINA1*, among which rs210222822 and rs41257068 presented in exons, rs207601878 presented in an intron, and rs208607693 was in the 5′-UTR. Analyses of pairwise D′ measures of linkage disequilibrium (LD) showed strong linkage among these four SNPs (D′ = 0.99–1.00), and a 9 Kb haplotype block involving three main haplotypes with GTGT, CCCC and CCGT was inferred. An association study revealed that all four single SNPs and their haplotypes had significant genetic effects on milk protein percentage, milk protein yield and milk yield (*P* = 0.0458 −  < 0.0001). The phenotypic variance ratio for all 11 significant SNP-trait pairs ranged from 1.01% to 7.54%. The candidate gene of *SERPINA1* revealed by our previous RNA-Seq study was confirmed to have pronounced effect on milk protein traits on a genome level. Two SNPs (rs208607693 and rs210222822) presented phenotypic variances of approximately 7% and may be used as key or potential markers to assist selection for new lines of cows with high protein concentration.

## Introduction

Bovine milk represents an essential source of nutrients for lactating calves and a key raw material for human food preparations (D’Alessandro, Zolla & Scaloni, 2011). As one of major nutrient components in milk, the concentration of bovine milk protein is closely related to the nutritive value of milk and milk selling price. In addition, bovine milk proteins also present important biological functions, such as, providing external nutrients and defense molecules against pathogens to suckling, directly stimulating the growth of neonate tissues/organs, and assisting animals to develop a proper immune system (D’Alessandro, Scaloni & Zolla, 2010). Studies have revealed that milk protein synthesis is regulated by a remarkable number of molecular cascades (Bonfatti et al., 2011; Gebreyesus et al., 2016; Sanchez et al., 2017; Schopen et al., 2009a; Schopen et al., 2009b). Thus, identification of the genes that are responsible for phenotypic variation in milk protein concentration is important to increase the understanding of milk protein synthesis and to enhance opportunities to improve milk protein composition in cattle. Our previous RNA-Seq study reported that *SERPINA1* gene was one of the most promising candidates to regulate milk protein concentration in dairy cattle (Li et al., 2016a). The direct evidence was the bovine mammary tissues collected from cows with high milk protein percentage had greater *SERPINA1* (Serpin peptidase inhibitor, clade A (Alpha-1 Antiproteinase, Antitrypsin), member 1) mRNA expression in comparison to the tissues collected from cows with low milk protein percentage (*q*-value = 1.17E−09) (Li et al., 2016a). The *SERPINA1* gene is located in BTA21 with a total length of 9,370 bp, containing 5 exons and 4 introns and encoding 416 amino acids (Khatib, 2005). Both bovine and human milk contains *α*1-antitrypsin (SERPINA1) (Beatty, Bieth & Travis, 1980), known as a potent serine protease inhibitor (Gettins, 2002), which inhibits several proteolytic enzymes, such as leukocyte elastase, trypsin, pancreatic elastase, chymotrypsin, collagenase, and plasmin (Dallas, Murray & Gan, 2015).

Therefore, the objectives of this study were to confirm the regulatory role of *SERPINA1* identified in our previous RNA-Seq study on milk protein traits from a genome level and further to uncover the genetic effects of *SERPINA1* on milk protein traits in Chinese Holstein population. We herein investigated polymorphisms of *SERPINA1* and their associations with five milk production traits, the linkage analyses among these identified polymorphisms in *SERPINA1* gene and the association analyses of haplotypes inferred with five milk production traits were also conducted.

## Materials & Methods

### Animal ethics

All protocols for collection of the blood and frozen semen samples of experimental individuals were approved by the Institutional Animal Care and Use Committee (IACUC) at China Agricultural University (Permit Number: DK996). The procedures for sampling, laboratory analysis, and data processing were following our previously published article (Li et al., 2019).

### Animal, phenotypes and traits

All samples and related data were collected from the cows from 17 farms of the Beijing Sanyuan Lvhe Dairy Farm Center (Beijing, China). A total of 1,027 Chinese Holstein cows from 17 sire families were involved in the present experiment with the family size ranging from 25 to 187 daughters with an average of 60 daughters per sire. All cows enrolled in the experiment were regularly tested using standard performance testing (Dairy Herd Improvement, DHI) and five milk production traits were recorded, including 305 d milk yield, 305 d protein yield, 305 d fat yield, average 305 d protein percentage, and average 305 d fat percentage.

The tubule frozen semen samples were collected for all 17 sires and prepared by Beijing Bull Station. Whole blood samples were collected from coccygeal vein of 1,027 Chinese Holstein cows using a 20-gauge BD Vacutainer needle (Beckton Dickinson, Franklin Lakes, NJ) and immediately stored at −20 °C prior to DNA extraction.

### Genomic DNA extraction

Genomic DNA was isolated from semen samples using standard phenol-chloroform procedures as described in previous study (Li et al., 2016b), whereas genomic DNA was extracted from blood samples using a TIANamp Blood DNA kit (TIANGEN Biotech, Beijing, China) following the manufacturer’s instructions. The quality and quantity of genomic DNA were measured using the gel electrophoresis and a NanoDrop 2000 spectrophotometer (Thermo Scientific, Hudson, DE, USA).

### Identification and genotyping of the SNPs

A total of 11 pairs of primers (Table S1) were designed using Primer3web Program (v.0.4.0) to amplify all exons, partial adjacent introns and 2,500 bp upstream of 5′ flanking sequences based on the genomic sequence of bovine *SERPINA1* gene referring to the UMD3.1 assembly (NCBI reference sequence accession no. AC_000178.1). A DNA pool was generated with genomic DNA from the 17 sires’ semen with 50 ng/µL/bull and was amplified with Polymerase Chain Reaction (PCR). Each PCR reaction consisted 50 ng genomic DNA, 0.5 µL of forward primer, 0.5 µL of reverse primer, 2.5 µL 10× PCR buffer, 2.5 mM dNTP, and 1 U of Taq DNA polymerase (Takara Biotechnology Co., Ltd., Dalian, China) with a total reaction volume of 25 µL. Thermal cycling conditions were: 94 °C for 5 min, followed by 35 cycles at 94 °C for 30 s, 56 °C for 30 s, and 72 °C for 30 s, a final extension at 72 °C for 7 min for all primers. PCR products were confirmed by gel electrophoresis on 2% agarose gels and by photography under UV light, and were bi-directionally sequenced by ABI 3730XL DNA analyzer (Applied Biosystems, Foster, CA, USA). And the sequences were aligned to the bovine reference sequences (UMD3.1) using BLAST (http://blast.ncbi.nlm.nih.gov/Blast.cgi) to identify potential SNPs.

The matrix-assisted laser desorption/ionization time of flight mass spectrometry assay (MALDI-TOF MS, Sequenom MassARRAY, Bioyong Technologies Inc. HK) was further applied for individually genotyping of the identified SNPs in 1,027 Chinese Holstein cows.

### Linkage disequilibrium (LD) analysis and haplotype construction

Based on the criterion of D′, the extent of linkage disequilibrium (LD) was measured between each pair of SNPs that were genotyped within the *SERPINA1* gene using the software Haploview (Barrett et al., 2005). Briefly, genotypes were imputed for each individual using the Beagle3.2 software program (Browning & Browning, 2007). After that, haplotype blocks with high LD of SNPs (D′ > 0.90) were defined based on confidence intervals methods (Gabriel et al., 2002). Haplotypes with frequencies >5% were considered distinguishable, whereas the haplotypes with relative frequencies <5% were pooled into a single group (Wang et al., 2013). Detailed procedures were described in our previously published research (Li et al., 2019).

### Statistical analysis

The effects of single SNPs or haplotypes in *SERPINA1* on five milk production traits were analyzed by the Mixed Procedure of SAS (SAS Institute Inc., Cary, NC) with the following model:

\begin{eqnarray*}{y}_{\text{ijklmn}}=\mu +{F}_{i}+Y{S}_{j}+{P}_{k}+b\times \mathrm{M}+{G}_{l}+{\alpha }_{m}+{e}_{\text{ijklmn}} \end{eqnarray*}

where, *y*_ijklmn_was the phenotypic value of each trait of cows (*n* = 1,027 for each trait);  *μ* was the overall mean; *F*_*i*_was the fixed effect of farm; *YS*_*j*_was the fixed effect of year-season; *P*_*k*_was the fixed effect of parity; M was the covariate effect of calving month; *b* was the regression coefficient of M; *G*_*l*_ was the fixed effect corresponding to the genotype of polymorphisms or haplotype; *α*_*m*_was the random polygenic effect, distributed as N (0, A ${\sigma }_{a}^{2}$), with the additive genetic relationship matrix A and the additive genetic variance ${\sigma }_{a}^{2}$; and *e*_ijklmn_ was the random residual, distributed as N (0, I ${\sigma }_{e}^{2}$), with identity matrix I and residual error variance ${\sigma }_{e}^{2}$.

The Bonferroni adjustment was used for single SNP and haplotype analyses according to the number of SNP loci or haplotype blocks. Associations were considered as significant if a raw *P* value < 0.05/N, where N is the number of SNP loci or haplotype blocks tested in analyses. Hardy-Weinberg equilibrium (HWE) tests were performed on each identified SNP. The SNP allele frequencies were calculated and the expected genotype numbers were estimated using the expected genotype frequencies under HWE. Chi-square analysis was used to compare the number of expected genotypes and observed genotypes, using 0.05 as the significance threshold value. The additive (a), dominance (d), and allele substitution (*α*) effects were estimated according to the equation proposed by Falconer & Mackay (1996), i.e.,  *a* = (AA − BB)∕2, *d* = AB − (AA + BB)∕2 and $\alpha =a+d \left( q-p \right) $, where AA and BB represent the two homozygous genotypes, AB is heterozygous genotype, and p and q are the allele frequencies of corresponding loci.

### Phenotypic variance

The effect of each individual SNP on a specific trait was measured as the proportion of phenotypic variance of the trait explained by the SNP. The proportion of variance explained by a SNP was calculated as follows,

\begin{eqnarray*}\text{Phenotypic variance ratio}=2p \left( 1-p \right) {\alpha }^{2}/{\sigma }_{p}^{2}. \end{eqnarray*}

Where, *p* is the allele frequency of the analyzed SNP, *α* is the average effect of gene substitution calculated using the linear mixed model, and ${\sigma }_{p}^{2}$ isthe estimate of the phenotypic variance using the complete DHI data of the Chinese dairy cattle population.

## Results

### Phenotype data

Descriptive statistics of five milk production traits from 1,027 Chinese Holstein cows were presented in Table 1. All phenotypic values of five milk production traits followed approximately normal distributions and were able to be used for the following association studies.

**Table 1:** Descriptive statistics of five milk production traits.

**Traits**	**Number**	**Average**	**Standard deviation**	**Coeff of variation**	**Maximum**	**Minimum**
Milk yield (kg)	1027	10441.46	2184.85	20.92	16,040	404
Milk fat yield (kg)	1027	365.92	83.49	22.82	632.20	19
Milk protein yield (kg)	1027	329.11	67.30	20.45	484.70	19.70
Milk fat percentage (%)	1027	3.53	0.51	14.52	7.50	0.74
Milk protein percentage (%)	1027	3.17	0.22	6.94	5.27	0.63

### SNPs identification

Through resequencing the entire coding sequences, partial adjacent introns and 2,500 bp upstream of 5′ flanking sequences, a total of four SNPs were identified for the *SERPINA1* gene. One (rs208607693) was located in the 5′ -UTR, one (rs207601878) was intronic and the other two (rs210222822 and rs41257068) were located in exonic regions (Table 2). Both exonic SNPs were synonymous substitutions. All four SNPs were in Hardy-Weinberg equilibrium (*P* > 0.05), and the locations and allele frequencies of the four SNPs were summarized in Table 3.

**Table 2:** Information for the four identified SNPs in *SERPINA1* gene.

CHR	RefSNP	SNP locus	Alleles	Location	Position	Gene
21	rs208607693	g.1164C > G	C/G	5′-UTR	59589061	*SERPINA1*
21	rs210222822	g.5608C > T	C/T	Exon-2	59582289	*SERPINA1*
21	rs41257068	g.5746G > C	G/C	Exon-2	59582151	*SERPINA1*
21	rs207601878	g.8123T > C	T/C	Intron-3	59579774	*SERPINA1*

**Table 3:** Genotypic and allelic frequencies and Hardy-Weinberg equilibrium test of four SNPs of *SERPINA1* gene in Chinese Holstein cattle.

**Position**	**Locus**	**Genotypes**	***N***	**Frequency**	**Allele**	**Frequency**	**Hardy-Weinberg equilibrium *χ*2 test**
5′ flanking region	rs208607693 g.1164C > G	CG	461	0.463	C	0.631	*P* > 0.05
		CC	397	0.399	G	0.369	
		GG	137	0.138			
Exon-2	rs210222822 g.5608C > T	CT	469	0.470	C	0.630	*P* > 0.05
		CC	394	0.395	T	0.370	
		TT	135	0.135			
Exon-2	rs41257068 g.5746G > C	CG	432	0.427	C	0.306	*P* > 0.05
		CC	93	0.092	G	0.694	
		GG	486	0.481			
Intron-3	rs207601878 g.8123T > C	CT	410	0.422	C	0.338	*P* > 0.05
		CC	124	0.128	T	0.662	
		TT	438	0.451			

### Associations between *SERPINA1* gene and milk production traits

Associations between the four SNPs of *SERPINA1* and five milk production traits are presented in Table 4. All four SNPs (rs208607693, rs207601878, rs210222822 and rs41257068) had significant (*P* = 0.0458 −  < 0.0001) associations with five milk production traits, with the exception that no significant associations were observed between two SNPs (rs208607693 and rs210222822) and milk fat percentage. Phenotypic variances (>1%) explained by the four SNPs in *SERPINA1* gene were observed in 11 significant pairs of SNP-trait, among which rs208607693 and rs210222822 presented phenotypic variances of approximately 7% in milk fat yield and protein yield.

**Table 4:** Associations of four SNPs of *SERPINA1* gene with milk production traits in Chinese Holstein cattle (LSM ± SE).

**Locus**	**Genotype**	**Milk yield**	**Fat yield**	**Fat percentage**	**Protein yield**	**Protein percentage**
rs208607693 g.1164C > G	CC(397)	10,556 ± 62.58^A^	373.61 ± 2.61^A^	3.570 ± 0.025	331.29 ± 1.90^A^	3.156 ± 0.009^A^
	CG(461)	10,576 ± 63.64^A^	372.06 ± 2.65^A^	3.569 ± 0.026	333.85 ± 1.93^A^	3.181 ± 0.009^B^
	GG(137)	10,185 ± 84.49^B^	354.09 ± 3.55^B^	3.597 ± 0.034	318.47 ± 2.59^B^	3.176 ± 0.012^AB^
	*P-value*	**<.0001**	**<.0001**	0.6728	**<.0001**	**0.0061**
	Variance	**5.10E−02**	**7.41E−02**	1.58E−03	**7.54E−02**	1.47E−03
rs210222822 g.5608C > T	CC(394)	10,499 ± 62.99^A^	370.27 ± 2.64^A^	3.572 ± 0.025	328.96 ± 1.92^A^	3.159 ± 0.009^a^
	CT(469)	10,525 ± 63.57^A^	368.33 ± 2.65^A^	3.563 ± 0.026	331.77 ± 1.93^A^	3.181 ± 0.009^b^
	TT(135)	10,150 ± 84.85^B^	352.46 ± 3.57^B^	3.603 ± 0.034	316.79 ± 2.61^B^	3.175 ± 0.012^ab^
	*P-value*	**<.0001**	**<.0001**	0.4538	**<.0001**	**0.0228**
	Variance	**4.58E−02**	**6.00E−02**	2.61E−03	**6.93E−02**	8.60E−04
rs41257068 g.5746G > C	CC(93)	10,299 ± 94.52^A^	373.66 ± 3.98^A^	3.686 ± 0.038^A^	325.64 ± 2.90^AB^	3.189 ± 0.013^a^
	CG(432)	10,619 ± 62.09^B^	374.65 ± 2.58^A^	3.615 ± 0.025^A^	330.79 ± 1.88^A^	3.174 ± 0.009^ab^
	GG(486)	10,424 ± 62.56^AB^	358.11 ± 2.61^B^	3.506 ± 0.025^B^	325.33 ± 1.90^B^	3.156 ± 0.009^b^
	*P-value*	**0.0055**	**<.0001**	**<.0001**	**0.0054**	**0.0100**
	Variance	**2.15E−02**	9.09E−03	**3.43E−02**	3.26E−03	9.95E−03
rs207601878 g.8123T > C	CC(124)	10,425 ± 86.17^ab^	375.68 ± 3.62^A^	3.686 ± 0.035^A^	326.82 ± 2.64^AB^	3.190 ± 0.012^a^
	CT(410)	10,585 ± 63.38^a^	371.48 ± 2.65^A^	3.595 ± 0.025^B^	330.06 ± 1.93^A^	3.171 ± 0.009^ab^
	TT(438)	10,325 ± 64.11^b^	358.64 ± 2.68^B^	3.525 ± 0.026^C^	326.08 ± 1.95^B^	3.161 ± 0.009^b^
	*P-value*	**0.0126**	**<.0001**	**<.0001**	**0.0029**	**0.0458**
	Variance	2.73E−04	**2.55E−02**	**3.74E−02**	5.98E−04	**1.01E−02**

**Notes.**

*P*-value refers to the results of association analysis between each SNP and milk production traits. Different letter (small letters: *P* < 0.05; capital letters: *P* < 0.01) superscripts (adjusted value after correction for multiple testing) indicate significant differences among the genotypes.

The additive, dominant and substitution effects of four SNPs of *SERPINA1* on five milk-production traits are shown in Table 5. There were significant (*P* < 0.05) additive and substitution effects of rs210222822 on milk protein percentage. Significant additive and substitution effects were also observed (*P* < 0.05) in rs41257068 on protein and fat percentages and observed (*P* < 0.05) in rs207601878 on fat yield, fat percentage and protein percentage. Significant dominant effects of rs20860769 on protein percentage, rs41257068 and rs207601878 on milk yield and protein yield were also observed (*P* < 0.05). In addition, significant additive, dominant and substitution effects of rs208607693 and rs210222822 on milk yield, fat yield and protein yield, rs41257068 on fat yield were observed (*P* < 0.05).

**Table 5:** Additive, dominant and allele substitution effects of the four SNPs associated with milk production traits of *SERPINA1* in Chinese Holstein.

**Locus**	**Genetic effect**	**Milk yield**	**Fat yield**	**Fat percentage**	**Protein yield**	**Protein percentage**
rs208607693 g.1164C > G	Additive	185.77^**^	9.76^**^	−0.0132	6.41^**^	−0.0099
	Dominant	205.38^**^	8.21^**^	−0.0143	8.97^**^	0.0154^*^
	Substitution	239.20^**^	11.91^**^	−0.0169	8.75^**^	−0.0058
rs210222822 g.5608C > T	Additive	174.94^**^	8.90^**^	−0.0153	6.09^**^	-0.0080^*^
	Dominant	199.98^**^	6.97^**^	−0.0249	8.90^**^	0.0137
	Substitution	226.53^**^	10.71^**^	−0.0218	8.39^**^	-0.0044^*^
rs41257068 g.5746G > C	Additive	−62.50	7.78^**^	0.0901^**^	−0.15	0.0164^*^
	Dominant	257.50^*^	8.77^**^	0.0194	5.30^**^	0.0016
	Substitution	−162.60	4.37^**^	0.0826^**^	−1.91	0.0158^*^
rs207601878 g.8123T > C	Additive	50.00	8.52^**^	0.0805^**^	0.37	0.0142^*^
	Dominant	210.00^*^	4.32	−0.0108	3.61^*^	−0.0042
	Substitution	−17.84	7.12^**^	0.0840^**^	−0.80	0.0156^*^

**Notes.**

The asterisk (*) means the additive, dominant or allele substitution effect of the locus indicate differ at *P* < 0.05 and the asterisk (**) means the additive, dominant or allele substitution effect of the locus indicate differ at *P* < 0.01.

### LD and haplotype analysis

**Figure 1:**
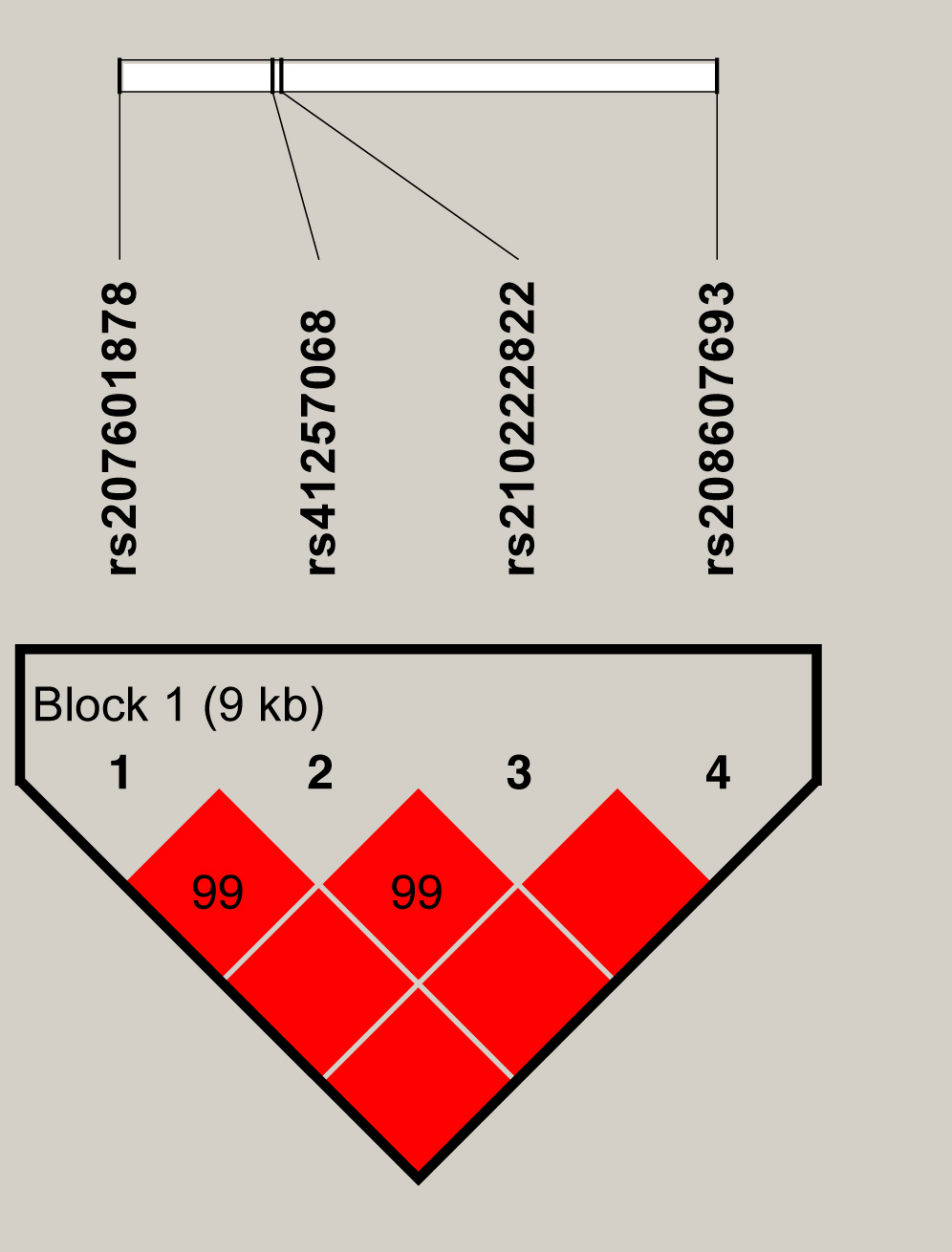
The haplotype blocks and pairwise linkage disequilibrium values (D′) for the four SNPs in *SERPINA1*. The values within boxes are pair wise SNP correlation (D′), bright red boxes without numbers indicate complete LD (D′ = 1). The brighter shade of red indicates higher linkage disequilibrium.

A markedly strong linkage (D′ = 0.99–1.00) was observed between the four SNPs in *SERPINA1*, as shown in Fig. 1. A 9 Kb haplotype block composed of four SNPs was inferred (Fig. 1) and three main haplotypes were formed. The common haplotypes GTGT, CCCC and CCGT occurred at the frequencies of 37.73%, 30.62% and 29.21% respectively (Table 6). Haplotype association analysis showed that haplotypes were highly associated with all five milk production traits (*P* = 0.0003 −  < 0.0001, Table 7).

## Discussion

In the present study, we explored the genetic variations of the *SERPINA1* gene and evaluated their associations with milk protein traits in Chinese Holstein cows, based on our previous RNA-Seq findings (Li et al., 2016a). Our results demonstrated significant genetic effects of the *SERPINA1* gene on milk protein traits on a genome level. This observation provided important SNP marker information that can be considered for genetic improvement in dairy breeding schemes.

Four identified SNPs were located in three different regions, including 5′-UTR, exon and intron. Of these, two SNPs (rs210222822 and rs41257068) in exonic regions are synonymous, which are not expected to change the function of affected proteins, as no substitution occurs as the amino acid level. However, increasing evidence indicates that synonymous SNPs could affect mRNA stability, therefore, impacting protein expression and function (Capon et al., 2004; Nackley et al., 2006). The association analyses in the current study suggest that these two synonymous SNPs are likely involved in the process of milk production through transcriptional regulation of the *SERPINA1* gene. Additionally, one SNP (rs208607693) located in 5′-UTR is markedly associated with milk production traits, because polymorphisms located in 5′-UTR of a gene may affect phenotypes through altering the promoter activity or transcription (Huang et al., 2013), it suggested that the genetic effects of SNP rs208607693 in 5′-UTR of *SERPINA1* gene on milk production traits was likely due to the impacts on its transcription. An intron does not hold a sequence for coding protein, but it is also important to regulate gene expression, regulation, transcription and mRNA splicing (Nott, Meislin & Moore, 2003). This is the likely reason that we observed the intronic variant (rs207601878) is also highly related to milk production traits. In addition, it is also probably that one of the four variants or another variant in the unsequenced region is in fact causative, which lead to the remaining variants with minor genetic effects showing highly significant associations with target phenotypes due to their strong LD with causative variant.

**Table 6:** Main haplotypes and their frequencies observed in *SERPINA1* gene.

***SERPINA1*haplotypes**	**rs208607693C > G**	**rs210222822C > T**	**rs41257068G > C**	**rs207601878T > C**	**Frequency(%)**
GTGT	G	T	G	T	37.73
CCCC	C	C	C	C	30.62
CCGT	C	C	G	T	29.21

**Notes.**

The Ref number of each SNP can be found in the haplotype Fig. 1.

**Table 7:** Haplotype associations of the four SNPs in *SERPINA1* with milk production traits in Chinese Holstein (LSM ± SE).

***SERPINA1*haplotypes**	**Milk yield**	**Fat yield**	**Fat percentage**	**Protein yield**	**Protein percentage**
H1H1(146)	10,197 ± 83.67^A^	352.47 ± 3.52^A^	3.582 ± 0.034^AC^	318.51 ± 2.56^A^	3.174 ± 0.012^A^
H2H1(241)	10,479 ± 73.87^B^	371.17 ± 3.09^BC^	3.604 ± 0.030^C^	331.09 ± 2.25^B^	3.189 ± 0.010^A^
H2H2(96)	10,459 ± 93.57^AB^	376.24 ± 3.93^B^	3.678 ± 0.038^C^	328.63 ± 2.87^B^	3.192 ± 0.013^A^
H2H3(166)	10,580 ± 78.33^B^	375.47 ± 3.29^B^	3.578 ± 0.031^AC^	332.20 ± 2.40^B^	3.154 ± 0.011^AB^
H3H1(236)	10,596 ± 74.78^B^	363.67 ± 3.13^CD^	3.493 ± 0.030^AB^	332.54 ± 2.28^B^	3.168 ± 0.011^A^
H3H3(92)	10,514 ± 102.14^AB^	353.30 ± 4.30^AD^	3.382 ± 0.041^B^	326.65 ± 3.13^AB^	3.110 ± 0.014^B^
*P-value*	**0.0003**	**<.0001**	**<.0001**	**<.0001**	**<.0001**

**Notes.**

*P*-value refers to the results of association analysis between each haplotype and milk production traits. Different letter (small letters: *P* < 0.05; capital letters: *P* < 0.01) superscripts (adjusted value after correction for multiple testing) indicate significant differences among the haplotypes. H1 = GTGT, H2 = CCCC, H3 = CCGT.

Generally, multi-SNPs association analyses involving multiple SNPs were considered more powerful than single SNP analysis (Akey, Jin & Xiong, 2001; Martin et al., 2000). Therefore, single SNP-based and haplotype association analyses were implemented in this study to determine the genetic effects of these variants on milk production traits. SNP-based association analysis indicated that all four tested SNPs were significantly associated with five milk production traits, except for rs208607693 and rs210222822 on milk fat percentage. Haplotype-based association analysis confirmed the results of single SNP analysis and provided further evidence for these associations. As *SERPINA1* was identified be a candidate for milk protein trait in our previous RNA-Seq study, the single SNP-based or haplotype-based analyses confirmed the significant associations between *SERPINA1* and milk protein traits, and further revealed the remarkable associations between *SERPINA1* and milk yield and milk fat traits. These results also supported that there is high genetic correlation between milk protein traits and milk yield and fat traits. Further functional studies are required to validate the functions of the *SERPINA1* gene.

Alpha-1-antitrypsin, a protease inhibitor encoded by *SERPINA1*, presents in human milk with relatively high concentration, as well as in bovine, porcine, and ovine colostrum. The concentration of alpha-1-antitrypsin gradually declines during the lactation period (Chowanadisai & Lonnerdal, 2002; Lonnerdal, 2010). Milk protease inhibitors play significant role in maintaining the level of other milk proteins via the partial inhibition of pancreatic proteases (Lonnerdal, 2003). Milk protease inhibitors present in human and animal milk are important for milk quality, therefore, infant health and development (Corley et al., 2017). For instance, alpha-1-antitrypsin in bovine milk may inhibit the hydrolysis of lactoferrin by trypsin (Khatib, 2005; Sinha, Bakhshi & Kirby, 1992). It has been reported that alpha-1-antitrypsin could reduce the susceptibility of mastitis in dairy cows by protecting lactoferrin from proteolytic degradation in mammary gland (Heihavand-Kheiripour et al., 2014). In addition, it was also observed that the mRNA expression of *SERPINA1* may affect milk composition and quality over the lactation curve (Chowanadisai & Lonnerdal, 2002; Matamala et al., 2017).

The SNPs rs210222822 and rs41257068 identified in this study were also found in North American Holstein population (at position 59307225 bp and 59307087 bp, GenBank accession number: X63129) (Khatib, Heifetz & Dekkers, 2005), individuals from Holstein-Friesian sires cohort (at position 59307225 bp and 59307087 bp, GenBank accession number: X63129) (Beecher et al., 2010), and small Chinese Holstein population (at position 59582289 bp and 59582151 bp, NCBI reference sequence accession no. AC_000178.1) (Guo et al., 2017; Li et al., 2010). Association analyses in this study revealed that SNP rs210222822 was significantly associated with three milk yield traits and milk protein percentage, which were partly confirmed by association with milk protein yield (Khatib, Heifetz & Dekkers, 2005), milk fat yield (Beecher et al., 2010; Khatib, Heifetz & Dekkers, 2005) and milk yield (Khatib, Heifetz & Dekkers, 2005; Li et al., 2010). A significant relationship between one identified SNP in *SERPINA1* gene and milk protein and fat percentage was reported in 408 Iranian Holstein cows (Heihavand-Kheiripour et al., 2014). However, this relationship was not observed in the present study, probably due to the different genetic background of animals. As is known, quantitative traits including milk protein traits are commonly affected by several causative genes and a great number of genes with minor effects, which are confirmed by aggregation the cow milk proteins reported in 20 recent proteomics publications producing an atlas of 4,654 unique proteins (Delosiere et al., 2019). A total of 59 proteins were exclusively detected in milk from early lactation, proposing six milk proteins as putative biomarkers of negative energy balance for dairy ruminants (Delosiere et al., 2019). Herein, *SERPINA1* gene is one of the key candidate genes regulating milk protein synthesis in dairy cows.

In our study, haplotypes were highly associated with all five milk production traits and haplotype CCCC had genetic merit for milk protein percentage. These results were consistent with previously published research, in which Khatib et al. (Khatib, Heifetz & Dekkers, 2005) found a *SERPINA1* haplotype, composed of five SNPs within exon regions, was associated with milk yield, fat yield and protein yield. Similarly, Beecher et al. (Beecher et al., 2010) reported a *SERPINA1* haplotype GCGGC had superior genetic merit for milk protein yield and milk fat percentage. Therefore, both previous studies and the present experiment demonstrated that *SERPINA1* gene is a promising candidate affecting milk production traits.

## Conclusions

In summary, we validated the significant associations of *SERPINA1* variants with milk protein traits. All SNPs that explained above 1% phenotypic variances could be used as potential marker for target traits selection. Specially, rs208607693 and rs210222822 are considered as key markers to be implemented in genomic schemes for improving milk protein concentration. However, further studies are needed to explore the function of *SERPINA1* on milk production and to confirm their importance to improve milk protein concentration.

##  Supplemental Information

10.7717/peerj.8460/supp-1 File S1 PCR primers information of *SERPINA1* gene **SNPs are detected.

10.7717/peerj.8460/supp-2 Supplemental Information 2 Data information of 1037 Chinese Holstein cattle for association analyses

10.7717/peerj.8460/supp-3 Supplemental Information 3 Reference sequence from forward primer sequence to reverse primer sequence for rs41257068

10.7717/peerj.8460/supp-4 Supplemental Information 4 Sequence for identifying SNP by PCR product sequencing for rs207601878

10.7717/peerj.8460/supp-5 Supplemental Information 5 Sequencing peaks showed in Chromas software for rs207601878

10.7717/peerj.8460/supp-6 Supplemental Information 6 Sequencing peaks showed in Chromas software for rs208607693

10.7717/peerj.8460/supp-7 Supplemental Information 7 Reference sequence from forward primer sequence to reverse primer sequence for rs207601878

10.7717/peerj.8460/supp-8 Supplemental Information 8 Reference sequence from forward primer sequence to reverse primer sequence for rs208607693

10.7717/peerj.8460/supp-9 Supplemental Information 9 Sequencing peaks showed in Chromas software for rs41257068

10.7717/peerj.8460/supp-10 Supplemental Information 10 Sequencing peaks showed in Chromas software for rs210222822

10.7717/peerj.8460/supp-11 Supplemental Information 11 Reference sequence from forward primer sequence to reverse primer sequence for rs210222822

10.7717/peerj.8460/supp-12 Supplemental Information 12 Sequence for identifying SNP by PCR product sequencing for rs210222822

10.7717/peerj.8460/supp-13 Supplemental Information 13 Sequence for identifying SNP by PCR product sequencing for rs208607693

10.7717/peerj.8460/supp-14 Supplemental Information 14 Sequence for identifying SNP by PCR product sequencing for rs41257068

## Additional Information and Declarations

### DNA Deposition

The following information was supplied regarding the deposition of DNA sequences: The genomic sequence of the bovine SERPINA1 gene refers to the UMD3.1 assembly:
GCF_000003055.6.

### Data Availability

The following information was supplied regarding data availability: Raw data and sequence data are available in the Supplementary Files.
