# Supplementary material for: SERPINA1 gene identified in RNA-Seq showed strong association with milk protein concentration in Chinese Holstein cows
Source: PeerJ. 2020 Feb 24;8:e8460. doi: 10.7717/peerj.8460 (PMC7045893; doi:10.7717/peerj.8460)
Supplement: File S1 — **SNPs are detected. [file peerj-08-8460-s001.docx]

**Supplemental file 1:**

**Table S1. PCR primers information of *SERPINA1* gene**

| **Primer** | **Location** | **Primer sequences (5’-3’)** | **Product size (bp)** | **Annealing Temp (℃)** |
| --- | --- | --- | --- | --- |
| *SERPINA1*-1 | 5’ flanking region | F-TCCTTCTGCCACAAGAACCT | 514 | 59 |
|  |  | R-GGTCTAAGGATGAGGGGAGC |  |  |
| *SERPINA1*-2 | 5’ flanking region | F-ACTCAGTAAGGGAAGCCTGG | 567 | 59 |
|  |  | R-AGGGTGGTAGTGTGGGATTG |  |  |
| *SERPINA1*-3 | 5’ flanking region | F-AACCCACTGAAAAGCCCAAG | **512**** | 59 |
|  |  | R-ACCTAAGGCTGATACGGGTG |  |  |
| *SERPINA1*-4 | 5’ flanking region | F-GTTTCAGAGAGATGGACCAGC | 437 | 58 |
|  |  | R-GGCAACTAACCCCATACAGC |  |  |
| *SERPINA1*-5 | 5’ flanking region | F-GTCACAGTCTCTAGGGCACA | 546 | 59 |
|  |  | R-CCCTGTCCTTCCCCGTATTT |  |  |
| *SERPINA1*-6 | Exon-1 | F-AAATACGGGGAAGGACAGGG | 483 | 59 |
|  |  | R-AACCTGGGCTACTCACTGTC |  |  |
| *SERPINA1*-7 | Exon-2 | F-TCCCTGCCATTGTTCTGAGT | **540**** | 59 |
|  |  | R-GGTTCAGGGTGTGGAGAAGA |  |  |
| *SERPINA1*-8 | Exon-2 | F-TCATCAGTCCAACACCAGCA | **550**** | 59 |
|  |  | R-TATCCCATTCTGTGCTGCCA |  |  |
| *SERPINA1*-9 | Exon-3 | F-GAGCCTGGTGAGCTACAGTC | 568 | 59 |
|  |  | R-ATGCATTCTGGCCTCTGTTG |  |  |
| *SERPINA1*-10 | Exon-4 | F-TCTGCAGGACAAGAGGATGG | **514**** | 59 |
|  |  | R-CACAACTTCCAAGCACGTGA |  |  |
| *SERPINA1*-11 | Exon-5 | F-GTGGATCAGACAGAGGCCTT | 518 | 59 |
|  |  | R-ATGCAAGAGGACGAAGGGTT |  |  |

Note: **SNPs are detected.
